# Viral Decoys: The Only Two Herpesviruses Infecting Invertebrates Evolved Different Transcriptional Strategies to Deflect Post-Transcriptional Editing

**DOI:** 10.3390/v13101971

**Published:** 2021-09-30

**Authors:** Chang-Ming Bai, Umberto Rosani, Xiang Zhang, Lu-Sheng Xin, Enrico Bortoletto, K. Mathias Wegner, Chong-Ming Wang

**Affiliations:** 1 Key Laboratory of Maricultural Organism Disease Control, Ministry of Agriculture, Qingdao Key Laboratory of Mariculture Epidemiology and Biosecurity, Yellow Sea Fisheries Research Institute, Chinese Academy of Fishery Sciences, Qingdao 266237, China; baicm@ysfri.ac.cn (C.-M.B.); zhxiang1997@126.com (X.Z.); xinls@ysfri.ac.cn (L.-S.X.); 2 Laboratory for Marine Fisheries Science and Food Production Processes, Pilot National Laboratory for Marine Science and Technology, Qingdao 266237, China; 3 Coastal Ecology Section, Alfred Wegener Institute Helmholtz Centre for Polar and Marine Research, Warden Sea Station, 25992 List auf Sylt, Germany; umberto.rosani@unipd.it (U.R.); Mathias.Wegner@awi.de (K.M.W.); 4 Department of Biology, University of Padova, 35121 Padova, Italy; enrico.bortoletto@phd.unipd.it; 5 College of Fisheries, Tianjin Agricultural University, Tianjin 300380, China

**Keywords:** PacBio SMRT, long-read sequencing, malacoherpesvirus, OsHV-1, HaHV-1, antisense transcription, ADAR editing, host defenses

## Abstract

The highly versatile group of Herpesviruses cause disease in a wide range of hosts. In invertebrates, only two herpesviruses are known: the malacoherpesviruses HaHV-1 and OsHV-1 infecting gastropods and bivalves, respectively. To understand viral transcript architecture and diversity we first reconstructed full-length viral genomes of HaHV-1 infecting *Haliotis diversicolor supertexta* and OsHV-1 infecting *Scapharca broughtonii* by DNA-seq. We then used RNA-seq over the time-course of experimental infections to establish viral transcriptional dynamics, followed by PacBio long-read sequencing of full-length transcripts to untangle viral transcript architectures at two selected time points. Despite similarities in genome structure, in the number of genes and in the diverse transcriptomic architectures, we measured a ten-fold higher transcript variability in HaHV-1, with more extended antisense gene transcription. Transcriptional dynamics also appeared different, both in timing and expression trends. Both viruses were heavily affected by post-transcriptional modifications performed by ADAR1 affecting sense-antisense gene pairs forming dsRNAs. However, OsHV-1 concentrated these modifications in a few genomic hotspots, whereas HaHV-1 diluted ADAR1 impact by elongated and polycistronic transcripts distributed over its whole genome. These transcriptional strategies might thus provide alternative potential roles for sense-antisense transcription in viral transcriptomes to evade the host’s immune response in different virus–host combinations.

## 1. Introduction

Viruses are the most abundant and diverse biological entities on the Earth [1]. With only a little of their own genetic information, they have evolved to exploit a high diversity of host species across the different branches of the tree of life, from bacteria to eukaryotes, causing diseases with significant impact on host populations [2,3]. Among these, the double stranded DNA (dsDNA) herpesviruses can infect a variety of hosts, but only two herpesviruses are known to infect invertebrates: Ostreid herpesvirus-1 (OsHV-1) and Haliotid herpesvirus-1 (HaHV-1) [4]. Both cause substantial losses for aquaculture of the Pacific oyster *Crassostrea gigas*, the blood clam *Scapharca broughtonii* (OsHV-1), and the abalone *Haliotis spp.* [5,6,7]. OsHV-1 and HaHV-1 are distantly related to the other herpesviruses, and constitute a new taxonomic family, *Malacoherpesviridae*, within the order Herpesvirales [4]. Although only roughly half of OsHV-1 and HaHV-1 genes are homologues [8], all Herpesvirales, and within them malacoherpesviruses, are comparatively similar in their genomic structure. Since herpesvirus transcription exploits the host’s cellular machinery, specific transcriptional mechanisms might play a decisive role in promoting successful viral replication. So far, for HaHV-1 and OsHV-1, transcriptomic approaches have focused almost exclusively on short-read sequencing in order to characterise the host’s antiviral responses [9,10,11,12,13,14]. Conventional short-read sequencing may provide useful information on gene expression, but this method does not capture full length mRNAs, preventing a complete understanding of the strategies that viruses use to exploit the host’s transcriptional machinery and circumvent antiviral defenses. The complex transcriptomic architectures typical of viruses, including nested isoforms and overlapping genes, can only be resolved with the recent introduction of long-read sequencing (LRS) technologies (single-molecule real-time (SMRT) and nanopore sequencing) [15,16,17]. Notably, this approach has revealed that as many as 2300 unique transcripts produced from the 79 genes of Herpes simplex virus type 1 (HSV-1) during in vitro infections [18,19] and has also been used to reveal variant transcripts and widespread antisense (AS) transcription in human cytomegalovirus [20]. In the dense-coding genomes of herpesviruses, AS transcription is quite frequent [21] and could play a functional role in controlling gene expression by RNA interference, chromatin remodeling, transcriptional interference, and RNA editing [22]. Since sense-antisense transcription creates double stranded RNAs, it could also play a role in post-transcriptional modifications performed by Adenosine deaminase acting on dsRNA-1 (ADAR1) genes of the host. This antiviral immune response that uses hyper-editing plays a physiological role in limiting the activation of Melanoma Differentiation Associated factor 5 (MDA5) by endogenous dsRNAs [23]. In addition, depending upon the virus–host combinations, ADAR1 can restrict or enhance viral replication [24]. The integration of viral genomic and transcriptomic data has suggested that RNA-directed antiviral responses mediated by ADAR1 explicitly target malacoherpesvirus RNAs, and that the coding genes of malacoherpesviruses have evolved to reduce the number of ADAR targets [25].

To characterize the transcriptional strategies of malacoherpesviruses facing ADAR-hyper-editing, we applied long-read RNA sequencing based on the PacBio SMRT technology to the small abalone *Haliotis diversicolor supertexta* infected with HaHV-1 and the blood clam *S. broughtonii* infected with OsHV-1. We reconstructed the HaHV-1 and OsHV-1 genomes using shotgun DNA sequencing and we traced the viral transcription along a time course of infection by Illumina RNA sequencing (RNA-seq). LRS data obtained from selected samples representing the exponential and stationary phase of each experimental infection were then produced and used to annotate the HaHV-1 and the OsHV-1 genomes with the corresponding transcriptional arrays, in order to untangle transcript dynamics and diversity and to decipher the detailed transcriptional strategies in vivo.

## 2. Materials and Methods

### 2.1. Experimental Challenges with Malacoherpesviruses

We infected *H. diversicolor supertexta* specimens by injecting a HaHV-1 homogenate obtained from abalones collected in Guangdong Province (China, 2003). Similarly, we used an OsHV-1 homogenate collected from infected blood clams (China, 2017) to infect *S. broughtonii* specimens. The infection trials were conducted for 60 and 72 h, respectively, with animal physiological conditions being monitored every 6–12 h. In detail, a pool of *H. diversicolor supertexta* (32.83–42.30 mm) were purchased and cultured for 17 days in tanks (30 L tanks, 1 L of water per individual) supplied with aerated, sand filtered sea water and fed with seaweed (*Laminaria japonica*); all tested negative to HaHV-1. The water was changed daily and maintained in the range of 17.4–18.9 °C. The viral inoculum was prepared with the pedal muscle of a *H. diversicolor supertexta* infected by HaHV-1-CN2003, as previously described [14] and 100 μL (adjusted at 10^4^ copies of viral DNA/μL) was injected into the pedal muscle. Paired negative controls were injected with seawater and maintained in separate tanks. *S. broughtonii* specimens were collected from a wild population (sizes: 56.48 to 68.74 mm) and were cultured for 24 days, fed with homemade shellfish diets, maintained at a temperature of 15.1–15.8 °C, and tested negative to OsHV-1. A viral inoculum was prepared from an infected blood clam collected in a local hatchery, as previously described [11] and 100 μL (adjusted at 10^4^ copies of viral DNA/μL) of the homogenate was injected into the foot of the clams, with paired controls injected with seawater.

### 2.2. Sample Collection, DNA, RNA Extraction, Library Preparation and Sequencing

**Figure 1:**
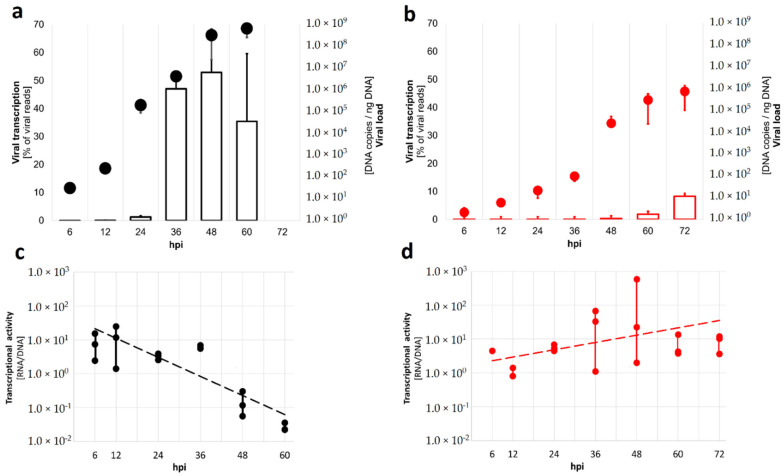
Malacoherpesvirus replication and transcription dynamics. The proportion of viral reads over total reads (viral transcription empty bars) and the viral replication measured by viral copies per ng of total DNA (viral load, dots referring to the secondary axis) are reported for HaHV-1 (black) (**a**) and OsHV-1 (red) challenges (**b**). The normalized transcriptional activity (i.e., the average virion transcription) expressed as the ratio of viral RNA over DNA showed distinct dynamics for both viruses with activity decreasing in HaHV-1 (**c**), while it was increasing for OsHV-1 (**d**) (ANOVA time × virus interaction F_1,9_ = 5.353, *p* = 0.046).

For both experimental infections, three samples were collected at 0, 6, 12, 24, 36, 48, 60 and 72 h post injection (hpi) (72 hpi only for OsHV-1 experiment). The abalone haemolymph was withdrawn from the cephalic arterial sinus located at the anterior part of the foot muscle using a 23-gauge needle attached to a 2 mL syringe. The clam hemolimph was collected from the adductor muscle sinus using a 23G needle attached to a 5 mL syringe. Since the amount of hemolymph from a single abalone was limited, four abalones were pooled per time point, while blood clams were harvested separately. The hemolymph samples were centrifuged at 800 rpm for 5 min at 4 °C and, after removing the supernatants, 1 mL of Trizol (Thermo Fisher Scientific, Waltham, MA, USA) was added to two of the three tubes and stored at −80 °C for RNA extraction. The remaining tube was directly stored at −80 °C for DNA extraction. DNA extraction was performed using a TIANamp™ Marine Animals DNA Kit (Tiangen Biotech, Beijing, China) according to the manufacturer’s protocol. The quantity and quality of the extracted DNA was measured with a NanoDrop^TM^ 2000 (Thermo Fisher Scientific). The quantification of HaHV-1 and OsHV-1 DNA loads in the collected samples was carried out by quantitative PCR (qPCR) [14]. For both experiments, the viral DNA constantly increased throughout the infection period and entered an exponential phase after 24 hpi for HaHV-1 or after 36 hpi for OsHV-1 (Figure 1). Total RNA was extracted using Trizol reagent kit, according to the manufacturer’s protocol. RNA quality was assessed on an Agilent 2100 Bioanalyzer (Agilent Technologies, Palo Alto, CA, USA).

One *H. diversicolor supertexta* and one *S. broughtonii* DNA sample was selected for DNA-seq. The library was constructed using a paired-end (PE) design with an insert size of 350 bp according to the manufacturer’s protocol (Illumina Inc., San Diego, CA, USA), and sequenced with an MiSeq instrument (Illumina). For *H. diversicolor supertexta* RNA-seq, mRNA was purified from total RNA using poly-T oligo-attached magnetic beads. The enriched mRNA was fragmented, and reverse transcribed into cDNA with random primers. Second-strand cDNA were synthesized by DNA polymerase I, RNase H, dNTPs, and appropriate buffer, purified with AMPure XP beads (Agencourt, Beverly, MA, USA), end repaired, poly(A) added, ligated to Illumina sequencing adapters, and selected using AMPure XP beads. The selected products were PCR amplified and sequenced with an Illumina HiSeq X with PE150 read layout. For *S. broughtonii* samples, rRNAs were removed and the remaining RNAs were fragmented by using fragmentation buffer and reverse transcribed into cDNA with random primers. Second-strand cDNA were synthesized, purified with QiaQuick PCR extraction kit (Qiagen, Venlo, The Netherlands), end repaired, poly(A) added, and ligated to Illumina sequencing adapters. Then UNG (Uracil-N-Glycosylase) was used to digest the second-strand cDNA. The digested products were size selected by agarose gel electrophoresis, PCR amplified, and sequenced using an Illumina HiSeq 4000 with PE150 read layout. For PacBio full-length cDNA sequencing, total RNAs of selected samples were reversely transcribed using the SMARTer PCR cDNA Synthesis Kit (Takara, Kyoto, Japan) according to the manufacturers’ protocol. Then, the second-strand cDNA were synthesized and amplified with Advantage^®^ 2 PCR Kit and recovered with AMPure PB beads. The quantities and sizes of the recovered PCR products were measured with Qubit 3.0 and Agilent 2100 Bioanalyzer, respectively, and mixed in equimolecular proportions. The mixed products were then DNA-damage, end-repaired and ligated to adapters using SMRTbell Template Prep Kit (PacBio, Menlo Park, CA, USA). The templates were digested with exonuclease and purified. Finally, the quality of the library was checked with Qubit 3.0 and Agilent 2100 Bioanalyzer and sequenced with a PacBio Sequel instrument.

### 2.3. Reconstruction of Malacoherpesvirus Genomes

Since the host genome is available only for *S. broughtonii*, different strategies have been adopted to reconstruct the HaHV-1 (HaHV-1-CN2003) and OsHV-1 (OsHV-1-CN2017) genomes. Reads were trimmed, allowing a minimal PHRED quality of 20 and removing sequencing adaptors. HaHV-1/*H. diversicolor supertexta* DNA-seq reads (331.5 M) were assembled de novo using the CLC assembler (CLC Genomic Workbench, Qiagen, Beverly, MA, USA), setting bubble and word sizes to “automatic” and the minimal contig length to 500 bp, obtaining 279,478 contigs. Reads were back mapped on the contigs to compute the local coverages and contigs were subjected to taxonomic classification against the nt NCBI database (*blastn*). Contigs identified as “Herpesvirales” were extracted and assembled into a consensus, using the contig coverages to identify repeated regions. The correctness of the consensus was evaluated by monitoring the paired read distance and the presence of unaligned read ends. For OsHV-1/*S. broughtonii* DNA-seq data, 83.7% of the trimmed reads mapped to the *S. broughtonii* genome [26] and were, therefore, removed. Unmapped reads (77M) were assembled into 85,951 contigs, and analyzed as described for HaHV-1. The genomic consensi of HaHV-1 and OsHV-1 were aligned with available references (AY509253, GQ153938, KP412538, KY242785, KY271630, MG561751, NC018874 and KU096999) using the *whole genome aligner* of CLC with the following parameters: minimum initial seed length = 10; allow mismatches in seeds = Yes; minimum alignment block length = 30. The Alignment Percentage (AP), which is the average aligned percentage of the genomes and the Average Nucleotide Identity (ANI), which is the percentage of exactly matching nucleotides for these aligned regions, were each computed. The phylogenetic tree was constructed using the Neighbor-Joining algorithm [27] based on ANI values. 

### 2.4. Annotation of the Viral Genomes and Definition of a New Gene Nomenclature

The *Iso-Seq* module of the SMRT Link software (PacBio) was used to group similar *ccs* into FLNC, i.e., joining multiple copies of the same transcript. A base correction approach of the FLNC consensus sequences was applied and only the FLNC sequences with accuracy greater than 99% were further analyzed. FLNC sequences covered at least by 10 *ccs* (h-FLNCs) were used for viral genome annotations. Following previous annotation strategies [6,28,29], we firstly annotated the HaHV-1 and OsHV-1 genomes with all the possible complete ORFs. Then, we exploited the h-FLNCs to extend these annotations to gene lengths, by mapping FLNCs using a splice-aware aligner (*large gap mapping tool*, CLC) applying 0.8 for length and 0.9 for similarity parameters. A manual annotation of viral genomes was performed to identify “Genes”, defined as the region covered by a FLNC cluster, and “transcripts”, defined as the most represented h-FLNC in each cluster, allowing multiple transcripts per gene in case of similarly represented h-FLNCs. In cases of polycistronic genes, both the polycistronic and the single ORF transcripts (if present) were annotated. Alternative transcripts were considered “*isoforms*” if they encoded the same ORF, while if they lost their coding potential were considered “*ncRNAs*”. We identified putative Transcript Starting Sites (TSSs) and Transcript Termination Sites (TTSs) along the viral genomes by searching for TATA boxes and polyadenylation signals (AAUAAA and AUUAAA) and visually assessing the change of local RNA coverages. We proposed a new gene nomenclature with the following composition: N(s/as)(-/p)(c/nc). “N” represented a number indicating the start position of the gene along the viral genome (in kb); “s” or “as” indicated whether the gene followed the forward (5′→3′) or reverse (3′→5′) genome orientation; “p” indicated polycistronic genes and “c” or “nc” indicated the presence or absence of a predicted ORF. The transcript nomenclature followed the same rules, adding the ORF name in case of multiple transcripts for a single gene. Finally, the ORF naming was kept consistent with the old nomenclature to facilitate further comparison.

### 2.5. Evaluation of Viral Transcription by RNA-seq

Trimmed RNA reads were mapped onto the reconstructed viral genomes (HaHV-1-CN2003 and OsHV-1-CN2017) or the viral transcripts extracted from the genomic annotations, applying 0.8 as threshold for both length and similarity(*CLC mapper*). The number of viral reads per sample was counted and divided by the viral DNA load to compute the normalized viral transcription levels per sample. Expression levels were computed for each transcript as Transcript Per Million (TPM). The maximum number of hits for a read was set to 10. If a read matched to multiple distinct transcripts the EM algorithm (CLC) assigned it to one of them by iteratively estimating the abundance of transcripts and assigning reads to transcripts according to these abundances.

### 2.6. Transcript Diversity and ADAR Hyper-Editing Analysis

To evaluate the overall transcript diversity, the FLNC sequences were clustered with Cd-hit [30] applying a 0.99 similarity threshold. FLNC rarefaction was performed by random sampling the sequences to a given depth. The *hyper-editing* tool [31] (https://github.com/hagitpt/Hyper-editing, accessed 1 January 2021) was applied with minimal modifications of the original version (e.g., *bwa*, SAMtools [32] and BEDTools [33] were implemented to overcome software incompatibilities). The tool parameters were adapted to our model, applying: 5 for Minimum of edited sites at Ultra-Edit read (%); 60 for Minimum fraction of edit sites/mismatched sites (%); 25 for Minimum sequence quality for counting editing event (PHRED); 60 for Maximum fraction of same letter in cluster (%); 20 Minimum of cluster length (%); and assuring that the hyper-editing clusters should not be completely included in the first or last 20% of the read. Outputs in BED format were parsed using custom scripts, and further analyzed using CLC Genomic Workbench v.21. Gene expression values computed as total mapped reads were used to normalize gene-specific hyper-editing levels. The level of strandedness of the libraries was evaluated by mapping the reads with strand constrains referred to the annotated genes (either “forward” or “reverse”) on HaHV-1, OsHV-1 and *S. broughtonii* genomes. Transcript strandedness levels were computed by the ratio of the number of “reverse” (expected sense of RNA sequencing) over “forward” mapped reads.

### 2.7. Single Nucleotide Polymorphism Analysis

To identify Single Nucleotide Polymorphisms (SNPs), the Illumina reads were mapped on the HaHV-1-CN2003 and OsHV-1-CN2017 genomes using a 0.8 length threshold and a 0.5 similarity threshold in order to allow the mapping of hyper-variable reads. SNPs were called with a cutoff of 1% of frequency, a minimal count of 10 and a minimal coverage of 100×. A quality threshold of PHRED30 for the variable position and of PHRED25 for the 5 nt window around the SNPs were used, and SNPs were sorted by type, counted and compared between samples. A-to-G and T-to-C SNPs were considered ADAR-compatible.

### 2.8. Statistical Analysis

A linear model of fitting time and viral species as predictors was analysed by ANOVA. Similarly, transcript lengths were analysed by ANOVA of a linear model fitting transcript class (gene, transcript, ORF) and viral species. Pairwise comparisons used Tukey’s HSD using the package *multcomp*. Correlations between hyper-edited reads and the number of viral reads were evaluated by Kendall’s tau (τ) coefficient analysis. The Raincloud plot was constructed with the R library *raincloudplots* [34], whereas all the analyses were performed using R 4.0.0 [35].

## 3. Results

The combination of short- and long-read HTS revealed that transcriptional strategies of HaHV-1 and OsHV-1 vary substantially, from replication and transcription dynamics to transcript diversity. Moreover, only few transcriptional features were shared by both viruses, suggesting that divergent transcriptional architecture characterizes malacoherpesvirus evolution and adaptation to different host taxa.

### 3.1. Virus Replication and Transcription Dynamics

We traced HaHV-1 and OsHV-1 transcription levels by Illumina RNA-seq on three biological replicates per time point along experimental infections (Tables S1 and S2). In small abalone, HaHV-1 became transcriptionally active at 24 h post injection (hpi) with the highest transcription levels at 36 and 48 hpi (Figure 1a), representing ~50% of all the sequenced reads in eight of nine samples from the latest time points (Table S2). In blood clam, the transcription of OsHV-1 started at 48 hpi, and only reached a peak of around 8% of the on-target reads (viral plus host reads) at 72 hpi (Figure 1b). Considering the transcriptional activity per virion, HaHV-1 showed a high transcriptional activity in the early times; however, transcriptional activity dropped substantially in the stationary phase with high viral loads (Figure 1c). OsHV-1, on the other hand, mildly increased its transcriptional activity during late infection stages (Figure 1d).

Based on Illumina RNA-seq data, we selected two HaHV-1 samples (H-36P, at 36 hpi and H-48P, 48 hpi) and two OsHV-1 samples (O-60P, 60 hpi and O-72P, 72 hpi) representing the exponential and the stationary phase of viral replication, respectively, for PacBio SMRT RNA sequencing (Table S2). For HaHV-1, the PacBio circular consensus sequences (*ccs*) could be condensed into 25,010 and 25,774 high-quality Full Length Non-Chimeric (FLNC) sequences for H-36P and H-48P, respectively (Table 1), with each FLNC representing a different and theoretically complete transcript. For OsHV-1, we obtained 17,074 and 22,388 high-quality FLNCs for O-60P and O-72P, respectively (Table 1). We could map 15.8 and 23.5% of the H-36P and H-48P FLNC sequences on the HaHV-1 genome, representing 50.4% and 75.7% of the total *ccs*, whereas a lower amount of FLNC sequences mapped to the OsHV-1 genome, representing 4.8 and 11.7% of the total *ccs* for O-60P and O-72P, respectively (Table 1). The viral mapping rates based on PacBio data mirrored viral transcription dynamics computed by Illumina RNA-seq data (Table 1).

### 3.2. Annotation of Malacoherpesvirus Genomes and Transcriptional Arrays

We sequenced the total DNA of one sample from each infection experiment and reconstructed HaHV-1 and OsHV-1 consensus genomes. A whole genome alignment of the 210.5 kb long HaHV-1-CN2003 genome together with the 203.1 kb long genome of OsHV-1-CN2017 and eight available references indicated a high similarity only within viral species. For example, HaHV-1-CN2003, “Taiwan” and “Victoria” HaHV-1 strains showed >97% of nucleotide similarity over >88% of aligned genome regions (Figure S1). Similarly, OsHV-1-CN2017 showed a considerable similarity (>99%) with the OsHV-1 references previously sequenced from blood clam hosts and a somewhat lower similarity (81–84%) with other OsHV-1 sequenced from infected oysters or scallops. HaHV-1 strains showed low nucleotidic similarities with OsHV-1 ones, e.g., 42% of similarity between HaHV-1-CN2003 and OsHV-1-CN2017 (Figure S1). The genomic architecture of all malacoherpesvirus genomes contained two unique regions (U_L_ and U_S_), each flanked by inverted repeats (TR_L_/IR_L_ and IR_S_/TR_S_), resulting in the following general architecture: TR_L_-U_L_-IR_L_-IR_S_-U_S_-TR_S_ (Figure S1). Structural differences between malacoherpesvirus genomes mainly arise from differences in the X region, which in OsHV-1 contains a unique gene (ORF115) with unknown function. In HaHV-1, the X region was missing completely, while the X region in OsHV-1-CN2017 was more than twice as long as in OsHV-1-µVars (3.8 kb versus 1.5 kb), which possesses two X regions with variation in their position compared to only one observed in the OsHV-1 reference and AVNV (Figure S1).

To annotate HaHV-1-CN2003 and OsHV-1-CN2017 genomes with “Genes”, “Transcripts” and “ORFs” we exploited only the FLNC sequences represented by at least 10 *ccs* (h-FLNC), thus removing poorly represented transcripts. The “Gene” annotations were inferred considering the region covered by a given cluster of FLNCs, while the most represented FLNCs within each cluster were annotated as “Transcripts”. “ORF” represented the coding regions identified bioinformatically, the latter being the only available gene annotations for extant malacoherpesvirus genomes as long as more detailed proteomic studies are missing. For some genes, where the longest transcript is also the most frequent one, “Gene” and “Transcript” annotations can match, while for other “Genes”, multiple “Transcripts” were annotated. The latter is often the case in polycistronic genes, where the polycistronic transcript joining multiple ORFs was often present with similar or lower frequencies than the transcripts encoding single ORFs. To reflect the transcriptional complexity of malacoherpesviruses, which was firstly reported by our data, we named the genes based on their position along the genome. The original nomenclature, which referred to ORFs only, was maintained as ORF naming (see M&M).

### 3.3. Gene Lengths Differ between HaHV-1 and OsHV-1

**Figure 2:**
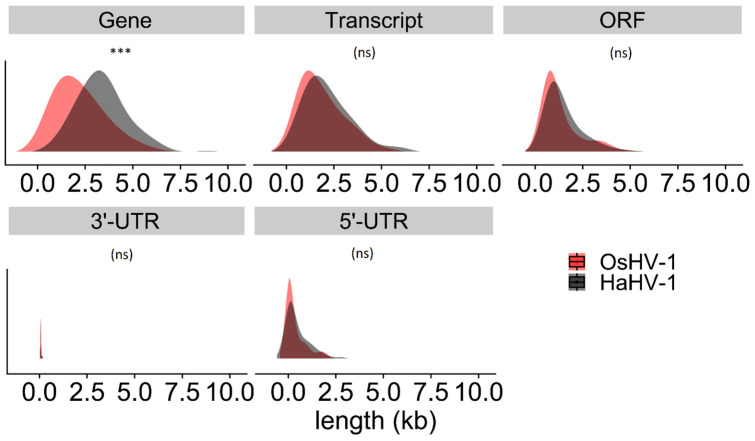
Density plots showing the distribution of the lengths of the annotated Genes, Transcripts, ORFs, 5′ and 3′-UTRs for HaHV-1 (black) and OsHV-1 genomes (red). While lengths of transcript, ORFs, 5′-UTR and 3′-UTR showed similar distributions among viruses, FLNC clusters (Genes) appeared significantly shorter for OsHV-1 (ANOVA virus*transcript type interaction F_2,596_ = 9.002, *p* < 0.001, asterisks denote significance level of pairwise Tukey post-hoc comparisons, ***: *p* < 0.001, ns: not significant, *p* > 0.05).

Using 704 h-FLNCs from H-36P and 1116 from H-48P we annotated the HaHV-1-CN2003 genome with 84 genes and 98 transcripts encoding for 113 ORFs (Table S3 and Supplementary Data). We could identify Transcription Starting Site (TSS) motifs at the 5′ of 46 genes and Transcription Termination Site (TTS) motifs at the 3′-end of 98 genes. We identified 30 polycistronic genes (35.7%), three transcripts encoding new ORFs and six non-coding RNAs (ncRNAs). The mean length of the HaHV-1 genes, inferred by FLNC clusters, was 3.44 kb, whereas the annotated transcripts had a mean length of 1.9 kb (Figure 2).

On average, the HaHV-1 5′-UTRs were 336 bp in length, whereas the 3′-UTRs were shorter with an average of 117 bp. The longest HaHV-1 gene, with a length of 8.9 kb, was the polycistronic *90.5aspc* gene encoding for ORF39–40–41. Up to 30% of the HaHV-1 genome is characterized by overlapping genes, with most of the overlaps characterized by genes encoded on opposite strands (27% of the genome length).

The OsHV-1 genome showed a similar number of genes, transcripts and ORFs. Based on 276 h-FLNC sequences from O-60P and 427 from O-72P, we annotated 94 genes, of which 27 were polycistronic (29.4%) and 111 transcripts encoding a total of 125 ORFs plus 3 ncRNAs (Table S3 and Supplementary Data). We identified 51 TSSs and 107 TTSs and we calculated that the average length of 5′-UTRs is 407 bp, compared to 112bp for 3′-UTRs. Because of the lower viral RNA coverage, we could not fully support some ORFs with PacBio data, including the DNA polymerase (ORF100). To reconstruct the DNA polymerase gene annotation, we used all FLNCs instead of using only the h-FLNCs. The mean length of the OsHV-1 genes (1.98 kb) were significantly shorter compared to HaHV-1 ones, whereas the mean lengths of the most represented transcripts and of the ORFs were not significantly different (Figure 2).

### 3.4. Transcript Diversity Differs between Malacoherpesviruses

Beside the static architecture based on the most represented FLNCs alone, we also investigated the transcriptional changes between exponential and stationary phases of the infection cycle by considering all the FLNCs.

Malacoherpesvirus transcript variability is generated both by alternative start and termination sites of transcription and by diversity at nucleotide level within transcripts. HaHV-1 had the highest transcript diversities at 48 hpi, with a 10-fold higher number of different transcripts (FLNCs) than OsHV-1 (Table 2). Higher coverage of viral RNA explained a major part of HaHV-1 transcript diversity. Rarefying HaHV-1 FLNCs to OsHV-1 levels, we observed a three-fold higher diversity for HaHV-1 (Table 2), suggesting that HaHV-1 transcription is characterized by a high number of low-frequency transcripts. Despite different transcriptional strategies between OsHV-1 and HaHV-1, shared transcriptional architectures affecting homologous genes were identified. The DNA polymerases (HaHV-1 *163.5sc*, 5.9 kb and OsHV-1 *147.5sc*, 5.6 kb), for example, are both 5′-flanked by genes encoded on the opposite DNA strand. These flanking genes are not conserved (HaHV-1 *162.4aspc* encoded a chloride channel protein, whereas OsHV-1 *147.0asc* a possible inhibitor of apoptosis), but both these genes are characterized by extended 5′-readtrough transcription (RTT) arching over the whole DNA polymerase gene, thus representing head-to-head antisense transcripts (Figure S2).

### 3.5. Gene Expression Trends Supported Different Transcriptional Strategies

**Figure 3:**
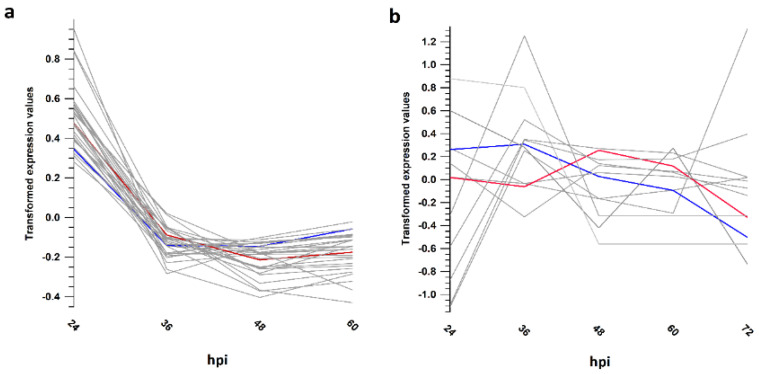
Expression trends analysis. The transcripts have been clustered in five groups based on their mean expression values per time point (Figure S3). (**a**) HaHV-1 cluster 3, including genes with decreasing expression trends along infection. (**b**) OsHV-1 cluster 3, including genes with decreasing expression trends along infection. The blue lines indicated the expression trend of the DNA polymerases, whereas the red lines indicated HaHV-1 *75.1sc* and OsHV-1 *30.5sc*.

Simultaneous transcription of most of the encoded genes was found in both viruses (Table S4), as already reported [36]. Transcript expression trend analysis highlighted that 46% of the HaHV-1 transcripts showed stable expression levels between 24 and 60 hpi (Figure S3, cluster 1); 30% showed decreased expression alongside infection, including two helicases, a DNA ligase, the DNA polymerase, the head-to-head antisense transcript associated to the DNA polymerase and *75.1sc* (Figure 3a and Figure S3, clusters 2 and 3), and 23% increased their expression levels, including a capsid protein and an envelope-fusion protein (Figure S3, cluster 5). Differently, 89% of the OsHV-1 transcripts showed an increasing expression from 24 to 72 hpi (Figure S3, clusters 1, 2, 4 and 5) and only 12 transcripts showed stable or decreasing expression trends (Figure 3b and Figure S3 cluster 3), including the DNA polymerase and *30.5sc*, a homolog of HaHV-1 *75.1sc*.

### 3.6. Tracing the Impact of ADAR1 Editing on Viral Transcripts

Malacoherpesvirus RNA variability at the nucleotide level was elevated by a large number of A-to-G conversions, possibly generated by post-transcriptional modifications mediated by ADAR1 in the form of hyper-editing [23]. ADAR1-compatible nucleotide substitutions (A-to-G and T-to-C) represented the majority of substitutions during productive infection (Figure S4). To support the presence of genuine ADAR editing, we evaluated the expression levels of ADAR1 in RNA-seq data, in relation to the level of hyper-editing of viral RNAs applying the *hyperediting* tool [31]. This tool identifies the reads with multiple variations, with A-to-G or T-to-C hyper-edited reads likely representing the enzymatic activity of ADAR1. 

**Figure 4:**
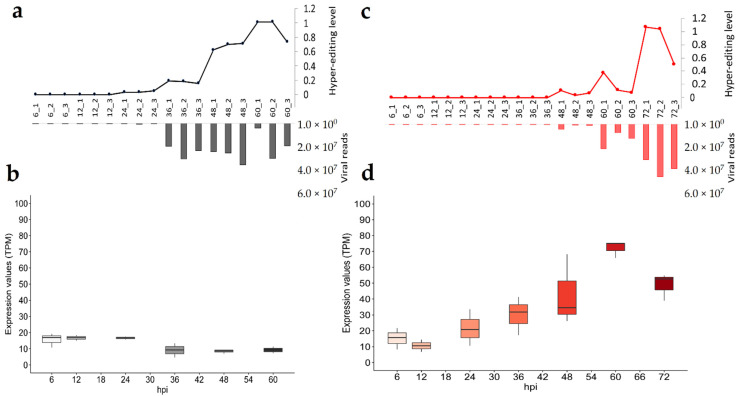
ADAR1 activity and expression levels during malacoherpesvirus infection. Along HaHV-1 (black data, (**a**)) and OsHV-1 (red data, (**c**)) infection experiments, lines represent the hyper-editing level calculated as hyper-edited reads over thousand mapped reads and bar plots represent the number of viral reads. Boxplots depict the expression of HdADAR1 (**b**) and SbADAR1 (**d**) as TPM.

*S. broughtonii* ADAR1 appeared upregulated from 36 hpi, reaching the maximal expression levels at 60 hpi (Figure 4d), compared to the stably expressed *H. diversicolor supertexta* ADAR1 (Figure 4b). However, expression differences did not match with the different hyper-editing levels, which increased with time in both infections. Hyper-editing reached its maximal level at late infection stages (average of 1 edited reads every 1000 mapped reads, Figure 4a,c, Table S2), and correlated with the amount of viral RNA (τ = 0.66 and 0.94 for HaHV-1 and OsHV-1, respectively). The number of hyper-edited reads mirrored the increase of ADAR-compatible SNPs along both infections. In total we found 2562 and 3062 different ADAR-compatible SNPs for HaHV-1 and OsHV-1, respectively, and most of them were sample-specific, sharing only a limited number of positions (Figure S4). During productive infection, the average SNP frequency is low in HaHV-1 (2%) and somewhat higher in OsHV-1 (10%, Figure S4). For HaHV-1, only two ADAR-compatible SNPs were recovered in the genomic SNPs, where they were present at frequencies around 1%. For OsHV-1, a higher genomic variability is present, which had a higher amount of non-ADAR SNPs among RNA SNPs (10–50% considering the 60 and 72 hpi samples) and also shared 59 ADAR-compatible SNPs with the genomic ones.

**Figure 5:**
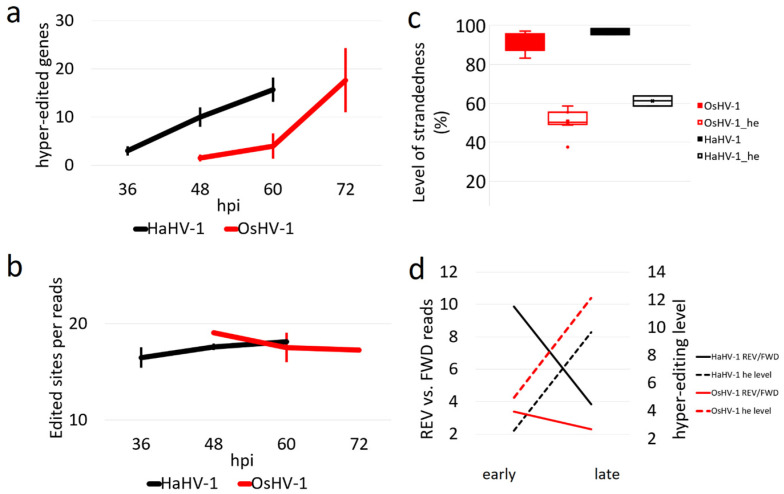
ADAR hyper-editing. (**a**) Box plot with the number of genes impacted by a minimal hyper-editing of 5‰. (**b**) Hyper-editing efficiency measured as the average number of edited sites per hyper-edited read. (**c**) Library strandedness computed considering either the bulk reads or the hyper-edited (he) reads mapped on the viral transcripts. (**d**) Comparison between the mean transcript strandedness and hyper-editing levels in early and late infection points for the genes showing at least 5 hyper-edited reads per 1000 mapped reads.

The viral genes impacted by host hyper-editing are mostly conserved between time points for each virus. Since transcript abundance and number of hyper-edited reads greatly differed between HaHV-1 and OsHV-1 (122,853 vs. 12,099 hyper-edited reads), we computed normalized gene hyper-editing levels and showed that ADAR1 impacted a similar number of genes of both viruses, increasing along infection (Figure 5a). Notably, the efficiency of hyper-editing, i.e., the number of edits in a hyper-edited cluster, is similar (18 edits) between viruses and stable along infection (Figure 5b).

Finally, we investigated the strandedness of hyper-editing. OsHV-1 samples were sequenced using stranded Illumina libraries, resulting in the correct strandedness of 94–99% of reads mapped on the host genome or 85–97% mapped to the OsHV-1 genome. Differently from the total reads, the hyper-edited reads showed a half-and-half strand distribution (Figure 5c). HaHV-1 libraries were not stranded and, therefore, we re-sequenced the H-36 and H-48 samples using stranded libraries, obtaining very similar HaHV-1 transcription rates, hyper-editing levels and strand distribution (Table S2). For both viruses, we showed that the impact of ADAR hyper-editing on transcripts increased along infection, while the level of transcript strandedness decreased for the same genes (Figure 5d).

## 4. Discussion

Revealing the transcript architecture of viral genomes is essential to understand virus biology [21,37]. This information is missing for the only known invertebrate herpesviruses, the malacoherpesviruses OsHV-1 and HaHV-1, where gene annotations are based on bioinformatic gene identification only, leaving out transcriptional complexity which goes beyond ORFs. We applied RNA LRS in vivo to characterize the transcript diversity in the exponential and stationary phase of virus replication in the small abalone *H. diversicolor supertexta* and in the blood clam *S. broughtonii*, both representing important viral hosts suffering from mortality events. Despite similarities in genome structure, gene, transcript and ORF contents, these viruses differed substantially in their transcriptional strategies, revealing that each virus–host combination evolved along unique trajectories on the level of transcriptome structure and expression dynamics. 

Firstly, HaHV-1 transcripts were much more abundant in abalones than OsHV-1 ones in clams, and often exceeded 50% of all reads within a sample. Such abundance, reported from HSV-1 infecting human dermal fibroblast in vitro [19], was never reported for malacoherpesviruses, which are usually characterized by low viral transcription rates [12,13,38]. Indeed, HSV-1 “host cell shutoff” is typical for a lytic infection [39] and is accompanied by an almost complete suppression of cellular protein synthesis [40]. Similar to HSV-1, HaHV-1 was reported to be a neurotropic virus [41,42], while OsHV-1 was never associated to neurotropism since it replicates in the connective tissue of the gills and mantle [43]. Along with its high levels, HaHV-1 transcription started earlier at 24 hpi, whereas OsHV-1 transcription only started at 48–60 hpi. Despite the HaHV-1 boost in abalone, the transcriptional activity per virion along infection increased only for OsHV-1. The delayed onset of OsHV-1 replication and its different transcription dynamics might reflect peculiar strategies employed to infect tissues with different immunogenicity (e.g., neurotropism). Alternatively, the different temperatures (18 °C for abalone and 15 °C for clam) we applied during infections could lead to differential infection dynamics [44,45]. These temperatures were, however, chosen to resemble the environmental conditions for which mortalities were observed, and are similar to the temperature threshold necessary for OsHV-1 to cause disease in oysters (16 °C) [46], whereas only much higher temperatures (24 °C) were shown to limit the progression of OsHV-1 infection [47]. It is therefore unlikely that infection dynamics during mortalities in both hosts differ substantially from our experimental infections.

A similar number of genes were found in the annotations of OsHV-1 and HaHV-1, although these have been based on the most represented transcripts only, and were supported by a different number of FLNC sequences. Polycistronism, which is common in herpesviruses and can regulate expression of distal genes within transcriptional interference networks (TIN) [48], was much more common in HaHV-1 than in OsHV-1. The TIN hypothesis suggested that the gene overlaps play a fundamental role in coordinating gene expression via the interactions between the transcriptional machineries of neighboring genes, thus resembling a novel layer of genetic regulation [48]. Indeed, in polycistronic genes, we always found distinct transcripts encoding the single ORFs next to the polycistronic transcript, suggesting that the functional role of polycistronism is not the expression of concatenated genes. Rather, polycistronism seems to be the result of viral transcript elongation in the stationary phase, mostly associated to 5′-RTT based on upstream TSSs and rarely impacting 3′-UTRs. Although AS transcription is often associated with 3’-UTR overlaps, an elongation of 5’-UTRs generating convergent overlaps was reported for cytomegalovirus [20]. This was the predominant situation in malacoherpesviruses, where 3’-UTRs appeared shorter compared to 5’-UTRs and generally not impacted by RTT. The propensity of miRNAs to target 3′-UTRs, and recent findings that miRNAs were differentially regulated during natural OsHV-1 infection of *Crassostrea gigas* [49], might suggest an adaptive benefit of shorter 3′-UTRs in escaping host defenses. For HaHV-1, 27% of the genome was predicted to contain AS gene overlaps, similar to what was reported for cytomegalovirus [20]. Arguably, full-length RNA approaches (e.g., PacBio SMRT) minimized the AS overestimation due to the analysis of partial transcripts and allowed an accurate estimation of Natural Antisense Transcripts (NATs). However, most of HaHV-1 and OsHV-1 head-to-head overlaps involved genes predicted to be coding, and should be considered as overlapping segments more than true NATs, as previously reported for cytomegalovirus using a similar approach [20]. In other herpesviruses the functions of single NATs have been often associated to the maintenance of latency and viral reactivation [50,51], but latency has not been shown for malacoherpesviruses thus far, which may suggest other functional roles for AS transcription. Indeed, one of the few shared features of both viruses is the presence of AS transcription along the DNA polymerase, generated by two non-homolog head-to-head AS transcripts showing similar expression trends as the DNA polymerases. Whether AS transcription is important for controlling the expression of the DNA polymerase needs further investigation. Alternatively, the function of AS transcription can potentially be linked to host ADAR-editing in the majority of cases. Hyper-edited reads and ADAR-compatible SNPs increased along the infection for both viruses. Abalone and blood clam ADAR1s showed high processivity even in comparison to the human counterpart [31] and edited both filaments of the dsRNAs, as shown by the balanced distribution between RNA strands of the hyper-edited reads. Similarly, the maximal hyper-editing frequencies (1.014 and 1.070‰ for HaHV-1 and OsHV-1, respectively) as well as the number of genes with a minimal hyper-editing frequency (5‰) were comparable between both viruses. This was unexpected since HaHV-1 produced 14-fold more RNAs than OsHV-1. However, we observed that HaHV-1 diffused the hyper-editing along almost the entire genome. This resulted from the extended antisense transcription of HaHV-1, which could possibly dilute the unfavorable effects of ADAR editing by reducing its per gene incidence. Differently, OsHV-1 exploited a balanced AS transcription of a limited number of genes to concentrate the hyper-editing in hotspots, possibly making them dedicated ADAR decoys. Controlling UTR lengths as well as the expression levels of sense–antisense gene pairs, viral transcription has the potential to diffuse or direct dsRNA formation and thereby influence RNA editing.

## 5. Conclusions

With the use of LRS we could unveil the complex transcriptional landscapes of malacoherpesviruses, which differ substantially between virus–host combinations. This complexity was generated by transcript elongation, polycistronism and frequent AS transcription, which would go unnoticed in conventional RNA-seq studies. Transcript elongation in HaHV-1 opened the door for ADAR1 hyper-editing, further increasing transcript diversity. With OsHV-1 limiting UTR elongation and concentrating ADAR edits, and HaHV-1 diversifying and diluting ADAR edits, we could thus observe divergent transcriptional strategies in response to post-transcriptional editing by the host. The functional significance of the different transcriptional strategies employed by the two malacoherpesviruses as well as revealing of the biological significance of ADAR1 hyper-editing represent challenges for future research, but this understudied functional role of AS transcription might also be exploited as a therapeutic target in other systems.

## Figures and Tables

**Table 1:** Viral RNA-seq analysis. The considered virus, the sample ID, the sequencing technology and depth, the number of PacBio *ccs* or Illumina reads, the number of *ccs* grouped into FLNC (FLNC-*ccs*) and the number of FLNC clusters are reported per sample. In parentheses are indicated the percentages of the FLNC-*ccs*, Illumina reads and FLNC clusters which mapped to the HaHV-1 or OsHV-1 genomes.

Virus	Sample	Technology	Seq. Depth	Total *ccs* or Reads	No. of FLNC-*ccs*	No. of FLNC
HaHV-1	H-36P	PacBio	20.0 Gb	257,457	218,594 (50.4%)	25,010 (15.8%)
	H-48P	PacBio	24.9 Gb	310,674	252,845 (75.7%)	25,774 (23.5%)
	H-36I	Illumina	6.8 Gb	45,723,904 (45%)	/	/
	H-48I	Illumina	8.3 Gb	55,518,872 (67%)	/	/
OsHV-1	O-60P	PacBio	38.1 Gb	550,552	383,717 (4.8%)	17,074 (7%)
	O-72P	PacBio	45.9 Gb	613,981	320,968 (11.7%)	24,378 (8.5%)
	O-60I	Illumina	14.4 Gb	83,222,092 (2.8%)	/	/
	O-72I	Illumina	11.6 Gb	55,015,939 (9.9%)	/	/

**Table 2:** Transcriptional dynamics. The number of different transcripts (similarity < 0.99), the diversity values calculated as the ratio between variable over total viral transcripts and the lengths of 5′- and 3′-UTRs were reported for the four PacBio samples. The percentages of UTR length increase between early and late time points were reported. In parentheses beside the number of different transcripts for the two HaHV-1 samples are also indicated the different transcripts obtained considering the HaHV-1 FLNCs rarefied to OsHV-1 levels.

Scheme ID	No. of Transcripts	Diversity Value	5′-UTRs	3′-UTRs
H-36P	1216 (417)	0.31	774	145
H-48P	1656 (636)	0.27	1359 (+76%)	194 (+34%)
O-60P	142	0.12	417	119
O-72P	188	0.09	436 (+5%)	139 (+17%)

## Data Availability

All the raw data discussed in this paper have been submitted to the NCBI SRA database with project ID shown in Table S1. The genomes of HaHV-1-CN2003 and of OsHV-1-CN2017 are submitted to NCBI under accession ID MW412419 and MW412420, and the corresponding annotation files are provided as Table S3.

## Supplementary Materials

The following are available online at www.mdpi.com/article/10.3390/v13101971/s1, Table S1: Details of the sequencing datasets produced for this study. Species, the sequencing approach and technology, the number of sequenced samples, the number of sequenced nucleotides, the downstream applications and the accession IDs in the NCBI SRA archive were reported. Table S2: Viral DNA and RNA quantifications. For each sample the ID, description, DNA quantification data (viral copies per ng of DNA, logarithmic transformation, average per time point and standard deviation) and RNA quantification data (viral reads, total reads, percentage of viral reads, average per time point and standard deviation) are reported. The samples in bold are the ones selected for PacBio RNA-seq. Exclusively for blood clam/osHV-1 datasets, the number and percentage of on-target reads was calculated counting only the reads mapped on the host and OsHV-1 genomes, instead of the total reads. The two abalone samples denoted by a “_S” in the ID column referred to the same RNA samples sequenced with stranded libraries. Column N indicated the hyper-editing level measured in these samples as number of hyper-edited reads over 1000 mapped reads. Table S3: HaHV-1 and OsHV-1 genome annotations (table). Table S4: Expression and hyper-editing. The expression levels as TPMs (log10) are reported for each transcript together with the cluster assigned in the expression trend analysis. For hyper-editing levels, the number of hyper-edited reads and the hyper-edited frequencies (computed as hyper-edited reads over 1000 mapped reads) were reported (red and green columns, respectively). Supplementary Data: HaHV-1 and OsHV-1 genome sequence and annotation files (compressed archive). Figure S1: Malacoherpesvirus genome reconstruction by DNA-seq. Figure S2: Overview of the transcriptional arrays of DNA polymerase of HaHV-1 (A) and OsHV-1 (B). a. Gene (blue) and ORF (yellow) reference annotations are indicated by arrows and labelled with the corresponding names. b. The transcriptional array based on the h-FLNCs is exemplified by lines showing the encoded CDSs as yellow arrows. Red lines depict transcripts in the antisense orientation, and green lines transcripts in the sense orientation. c. Coverage profile based on all the FLNC sequences mapped in the antisense (red) or sense (green) orientations. Red arrows along the genome sequences represented polyadenylation signals. Figure S3: Trend analysis. HaHV-1 (A) and OsHV-1 (B) transcripts were clustered by transformed expression levels in five clusters. Figure S4: SNP analysis.
