# Supplementary material for: Viral Decoys: The Only Two Herpesviruses Infecting Invertebrates Evolved Different Transcriptional Strategies to Deflect Post-Transcriptional Editing
Source: Viruses. 2021 Sep 30;13(10):1971. doi: 10.3390/v13101971 (PMC8537636; doi:10.3390/v13101971)
Supplement: Supplementary file 1 [file viruses-13-01971-s001.zip › Figure S1.pdf]

Supplementary Information 3. Malacoherpesvirus genome reconstruction by DNA-seq.

Level of similarity between HaHV-1-CN2003, OsHV-1-CN2017 and 8 available Malacoherpesvirus genomes. Similarities were computed as Average Nucleotide Identity (ANI, upper comparison) and Alignment Percentage (AP, lower comparison).

List of considered genomes:

AY509253_2005	AY509253	Ostreid herpesvirus 1
GQ153938_ANVN	GQ153938	Chlamys acute necrobiotic virus
KP412538_China	KP412538	Ostreid herpesvirus 1
KU096999_Taiwan	KU096999	Abalone herpesvirus Taiwan/2005
KY242785_uVAR	KY242785	Ostreid herpesvirus 1
KY271630_uVAR	KY271630	Ostreid herpesvirus 1
MG561751_Italy	MG561751	Ostreid herpesvirus 1
NC018874_Victoria	NC_018874	Abalone herpesvirus Victoria/AUS/2009
OsHV-1-CN2017	this paper	Ostreid herpesvirus 1
HaHV-1-CN2003	this paper	Abalone herpesvirus

		1	2	3	4	5	6	7	8	9	10
NC018874_Victoria	1		43.02	42.64	42.38	42.38	42.84	41.85	98.70	41.86	97.69
GQ153938_ANVN	2	35.67		99.72	99.60	99.60	99.63	99.36	43.96	99.38	43.15
AY509253_2005	3	35.47	84.37		99.73	99.73	99.76	99.48	43.64	98.76	42.86
KY242785_uVAR	4	37.65	82.91	83.01		99.96	99.96	99.11	43.38	98.40	42.20
KY271630_uVAR	5	37.66	82.87	82.34	86.67		99.96	99.11	43.37	99.13	42.20
MG561751_Italy	6	36.81	79.56	79.00	82.31	82.31		99.58	44.33	99.61	43.08
KP412538_China	7	39.07	84.60	84.02	85.39	85.39	82.49		43.05	99.94	42.14
KU096999_Taiwan	8	89.29	36.33	36.22	38.10	38.09	37.19	39.52		42.93	99.60
OsHV-1-CN2017	9	38.70	84.40	83.26	84.64	84.62	81.71	99.01	39.43		42.04
HaHV-1-CN2003	10	88.95	35.76	35.67	37.71	37.70	36.84	39.11	91.02	39.01	

Neighbor Joining tree based on whole genome alignment (ANI values) of Malacoherpesvirus genomes.

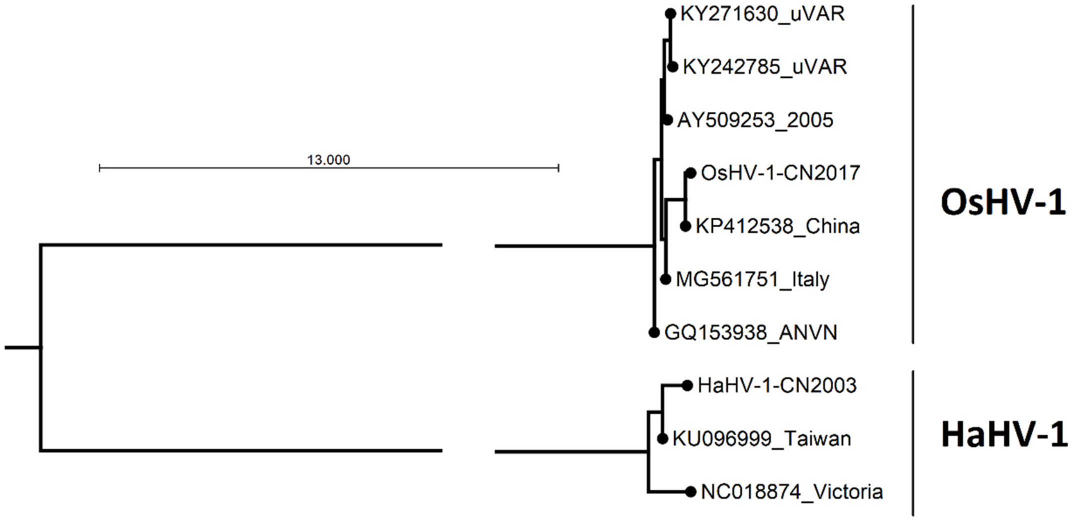

Comparison of the malacoherpesvirus genomic structures with archetypical herpesviruses.

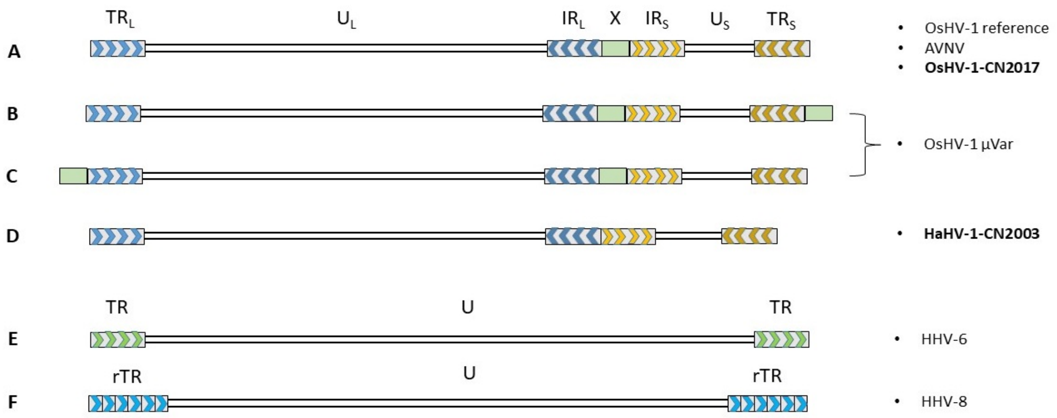

There are 4 typical sequence arrangements of herpesvirus genomes[1] (S. Figure 1). OsHV-1 genomes are composed of relatively complex elements, including two unique regions (U_L/U_S) that flanked by two pairs of inverted repeats (TR_L/IR_L and TR_S/IR_S) and separated by the X region (S. Figure 1, group A, B and C)[2]. Similar genomic architecture were found in variants infected Chinese scallops (AVNV)[3] and blood clam (OsHV-1-SB)[4], with the exception that X region was not detected in OsHV-1-SB. Since OsHV-1-SB genome was resolved through a PCR-based approach with primers designed based on OsHV-1 reference, we cannot exclude the possibility that the X region existed in OsHV-1-SB but missed due to variation in primer locus. Using HTS DNA-seq, we reanalyzed the genome sequence of OsHV-1 infecting S. broughtonii (OsHV-1-CN2017) and found a similar genome structure with a X region of 3.8kb, considerably longer than typical OsHV-1 X region (1.5kb). Two studies on OsHV-1-µVar genomes suggested that there are two copies of X along the genome[5,6]. Despite the variations, the genomic structure of OsHV-1 resembled that of human herpesvirus 1 (family *Herpesviridae*, subfamily *Alphaherpesvirinae*) and human herpesvirus 5 (HHV-5; family *Herpesviridae*, subfamily *Betaherpesvirinae*)[1]. The genome structure of HaHV-1-CN2003 is relatively simple, designed as TR_L–U_L–IR_L–IR_S–U_S–TR_S (Figure 1, group D), also resembling that of human herpesvirus 1 (subfamily *Alphaherpesvirinae*). There other classes of genomic structures are characterized by a unique sequence flanked by a direct repeat, e.g. human herpesvirus 6 (family *Herpesviridae*, subfamily *Betaherpesvirinae, S.* Figure 1, group E) or by a single unique sequence flanked by a variable number of repeated sequences at each terminus, e.g. human herpesvirus 8 (family *Herpesviridae*, subfamily *Alphaherpesvirinae, S.* Figure 1, group F).

1. Lefkowitz, E. J. *et al.* Virus taxonomy: the database of the International Committee on Taxonomy of Viruses (ICTV). *Nucleic Acids Res* **46**, D708–D717 (2018).
2. Davison, A. J. *et al.* A novel class of herpesvirus with bivalve hosts. *J. Gen. Virol.* **86**, 41–53 (2005).
3. Ren, W. *et al.* Complete genome sequence of acute viral necrosis virus associated with massive mortality outbreaks in the Chinese scallop, Chlamys farreri. *Virol. J.* **10**, 110 (2013).
4. Xia, J., Bai, C., Wang, C., Song, X. & Huang, J. Complete genome sequence of Ostreid herpesvirus-1 associated with mortalities of Scapharca broughtonii broodstocks. *Virol J* **12**, (2015).
5. Abbadi, M. *et al.* Identification of a newly described OsHV-1 µvar from the North Adriatic Sea (Italy). *J. Gen. Virol.* (2018) doi:10.1099/jgv.0.001042.
6. Burioli, E. a. V., Prearo, M. & Houssin, M. Complete genome sequence of Ostreid herpesvirus type 1 µVar isolated during mortality events in the Pacific oyster Crassostrea gigas in France and Ireland. *Virology* **509**, 239–251 (2017).
