# Supplementary material for: Viral Decoys: The Only Two Herpesviruses Infecting Invertebrates Evolved Different Transcriptional Strategies to Deflect Post-Transcriptional Editing
Source: Viruses. 2021 Sep 30;13(10):1971. doi: 10.3390/v13101971 (PMC8537636; doi:10.3390/v13101971)

Supplementary Information 6. Overview of the transcriptional arrays of DNA polymerase of HaHV-1 (**A**) and OsHV-1 (**B**). **a.** Gene (blue) and ORF (yellow) reference annotations are indicated by arrows and labeled with the corresponding names. **b.** The transcriptional array based on the h-FLNCs is exemplified by lines showing the encoded CDSs as yellow arrows. Red lines depicted transcripts in the antisense orientation, while green lines transcripts in the sense orientation. **c.** Coverage profile based on all the FLNC sequences mapped in the antisense (red) or sense (green) orientations. Red arrows along the genome sequences represented polyadenylation signals.

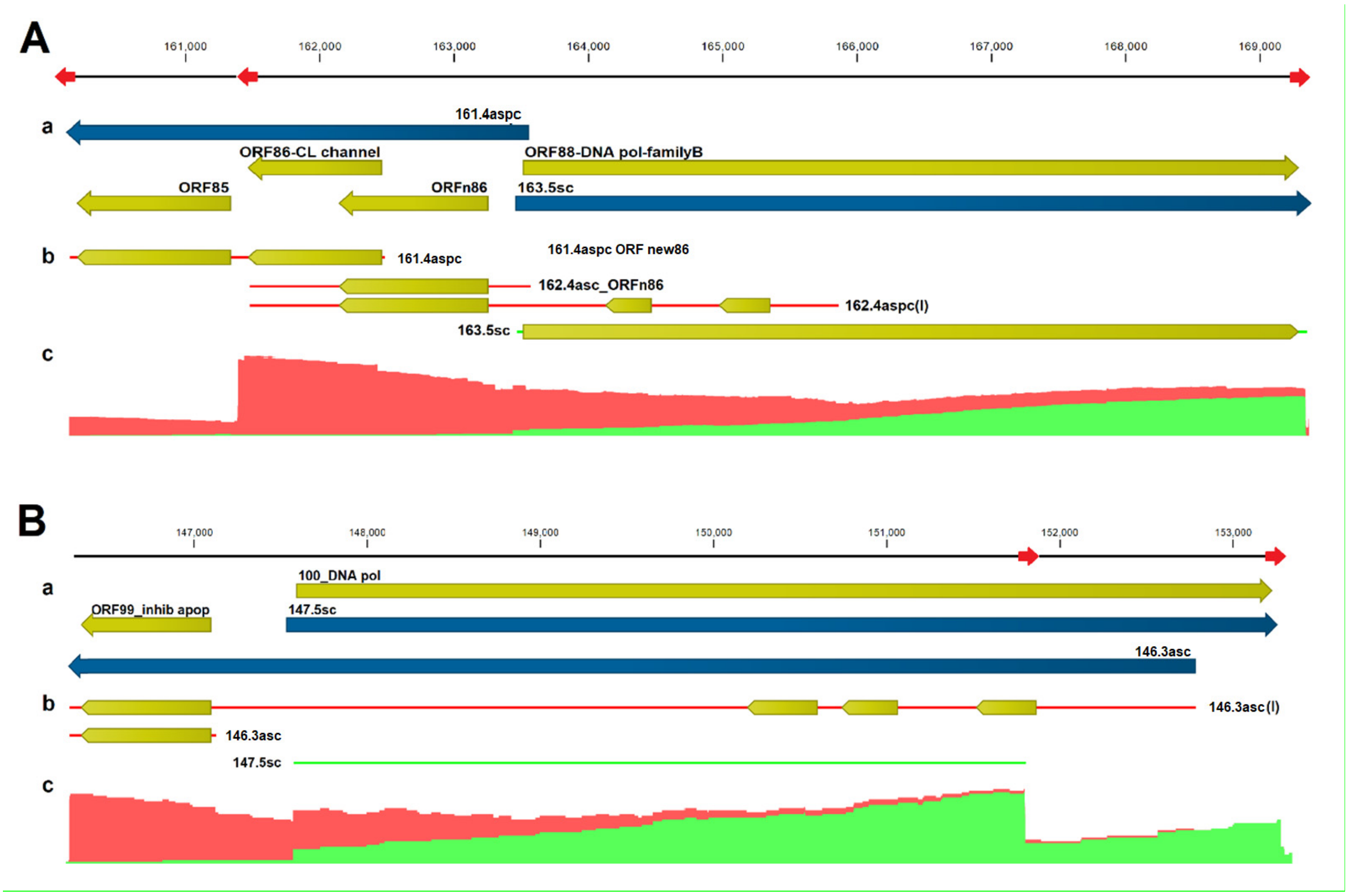

Supplement: Supplementary file 1 [file viruses-13-01971-s001.zip › Figure S2.pdf]
