# Supplementary material for: Viral Decoys: The Only Two Herpesviruses Infecting Invertebrates Evolved Different Transcriptional Strategies to Deflect Post-Transcriptional Editing
Source: Viruses. 2021 Sep 30;13(10):1971. doi: 10.3390/v13101971 (PMC8537636; doi:10.3390/v13101971)
Supplement: Supplementary file 1 [file viruses-13-01971-s001.zip › Figure S4.pdf]

Supplementary Information 9. SNP analysis.

The Number of viral SNPs are reported for HaHV-1 (**a**) and OsHV-1 (**b**). Black boxes represented SNPs other than A-to-G and T-to-C (shown as white boxes). For the HaHV-1 graph, the samples denoted by a "_S" are the re-sequenced samples with stranded library layout. The red vertical lines separated logarithmic and stationary phases of viral replication. The raw data are included in the table below.

The conservation of the variable positions is reported for the 2,561 HaHV-1 ADAR-compatible SNP (**c**) and for the 3,062 OsHV-1 ones (**d**).

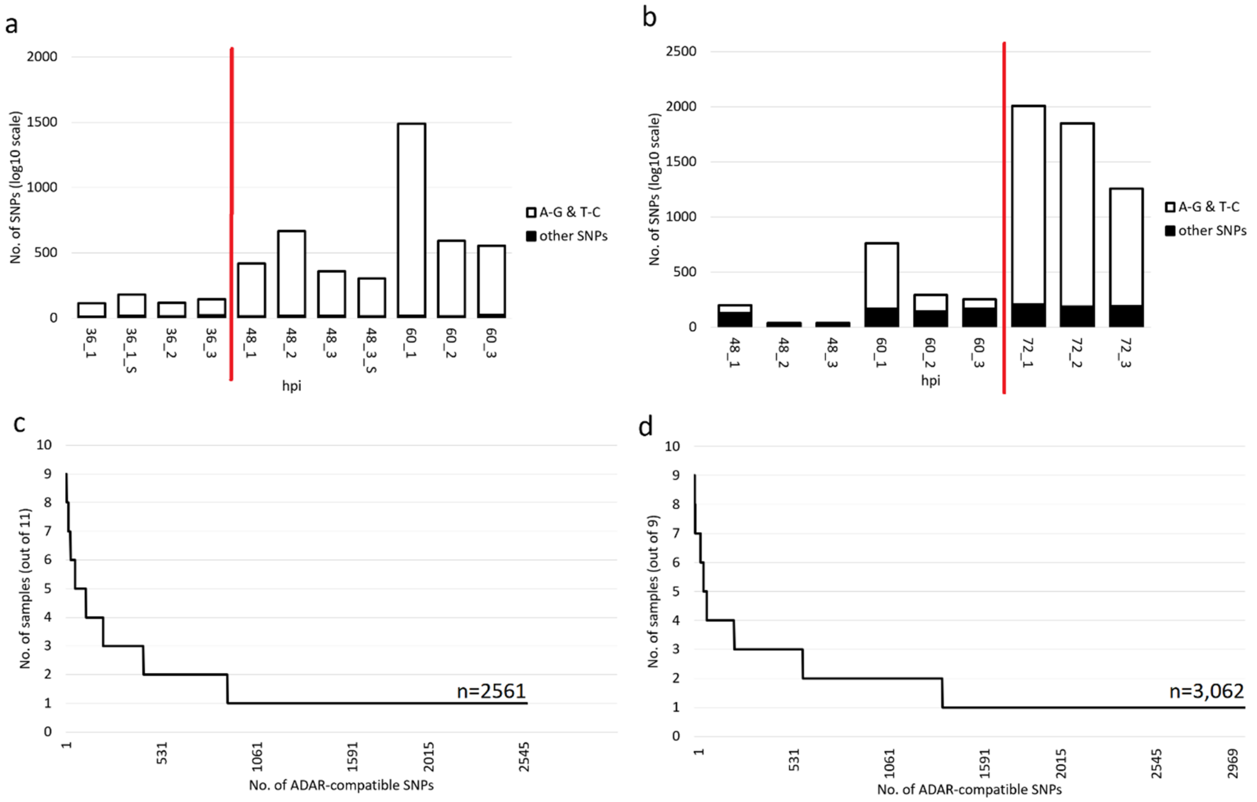

---

e. Data table. The number of total SNPs, other SNPs, ADAR-compatible SNPs, the mean frequency of ADAR SNPs and the percentages of ADAR-compatible SNPs over total SNPs are reported.

HaHV-1	total SNP	other	ADAR-SNP	freq	% of ADAR-SNP
6_1					
6_2	1	1			
6_3					
12_1					
12_2					
12_3					
24_1	1	1			
24_2	11	7	4		36,36
24_3	7	6	1		14,29
36_1	115	10	105	4,8	91,30
36_1_S	179	17	162	3,23	90,50
36_2	116	15	101	4,35	87,07
36_3	143	21	122	3,7	85,31
48_1	416	14	402	1,95	96,63
48_2	664	18	646	2	97,29
48_3	358	16	342	2,12	95,53
48_3_S	303	13	290	2,4	95,71
60_1	1489	16	1473	2,17	98,93
60_2	591	15	576	1,92	97,46
60_3	553	23	530	2,07	95,84

OsHV-1	total SNP	other	ADAR-SNP	freq	% of ADAR-SNP
6_1					
6_2					
6_3					
12_1					
12_2					
12_3					
24_1					
24_2					
24_3					
36_1					
36_2					
36_3					
48_1	203	129	74	25,4	36,45
48_2	36	33	3	23,8	8,33
48_3	41	34	7	23,5	17,07
60_1	762	172	590	7,2	77,43
60_2	294	145	149	15,7	50,68
60_3	255	169	86	26,8	33,73
72_1	2008	209	1799	3,73	89,59
72_2	1850	190	1660	3,6	89,73
72_3	1257	191	1066	5,08	84,81
