# Supplementary material for: Viral Decoys: The Only Two Herpesviruses Infecting Invertebrates Evolved Different Transcriptional Strategies to Deflect Post-Transcriptional Editing
Source: Viruses. 2021 Sep 30;13(10):1971. doi: 10.3390/v13101971 (PMC8537636; doi:10.3390/v13101971)
Supplement: Supplementary file 1 [file viruses-13-01971-s001.zip › Table S1.pdf]

Supplementary Information 1. Details of the sequencing datasets produced for this study. Species, the sequencing approach and technology, the number of sequenced samples, the number of sequenced nucleotides, the downstream applications and the accession IDs in the NCBI SRA archive were reported.

Species	Approach	Technology	No. of samples	No. of sequenced bases	Application	Accession ID (SRA archive)
H. diversicolor (gastropod) infected with HaHV-1	DNA-seq	Illumina, 2x350 bp	1	116 Gb	HaHV-1 genome assembly	PRJNA679801
	RNA-seq	Illumina, 2x150 bp	20	168 Gb	Evaluation of HaHV-1 transcriptional levels	PRJNA592737
	RNA-seq	PacBio, SMRT	2	44.9 Gb	HaHV-1 transcriptome annotation and transcriptional dynamics	PRJNA592736
S. broughtonii (bivalve) infected with OSHV-1	DNA-seq	Illumina, 2x350 bp	1	166 Gb	OsHV-1 genome assembly	PRJNA679800
	RNA-seq	Illumina, 2x150 bp	21	338 Gb	Evaluation of OsHV-1 transcriptional levels	PRJNA628528
	RNA-seq	PacBio, SMRT	2	83.9 Gb	OsHV-1 transcriptome annotation and transcriptional dynamics	PRJNA628527
