# Supplementary figures and images for: Viral Decoys: The Only Two Herpesviruses Infecting Invertebrates Evolved Different Transcriptional Strategies to Deflect Post-Transcriptional Editing
Source: Viruses. 2021 Sep 30;13(10):1971. doi: 10.3390/v13101971 (PMC8537636; doi:10.3390/v13101971)

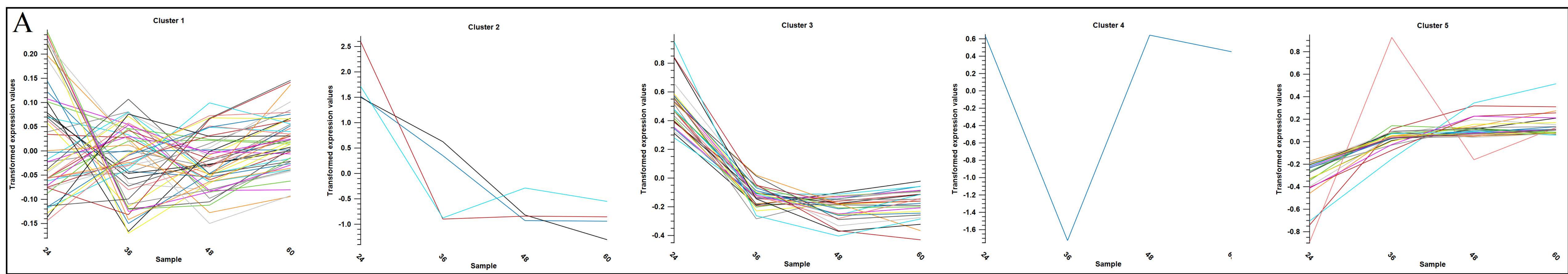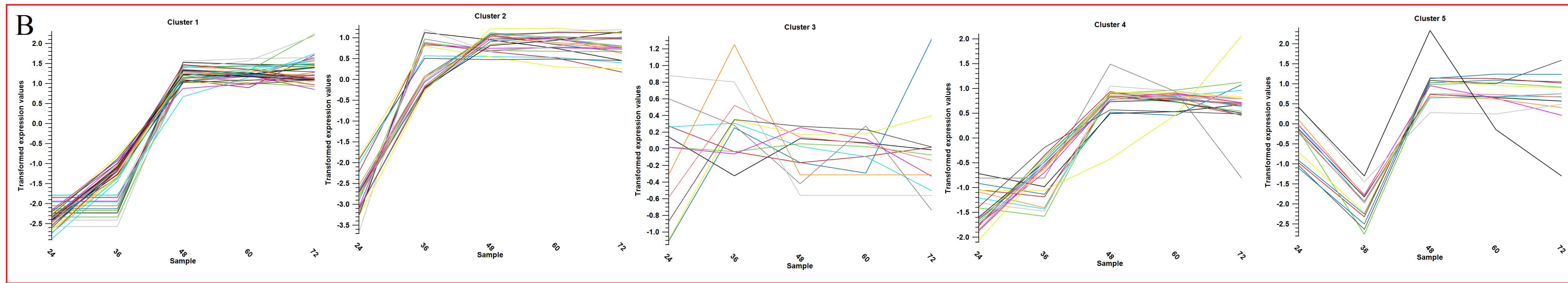

Supplement: Supplementary file 1 [file viruses-13-01971-s001.zip › Figure S3.pdf]
